# Assessment of a New Nanostructured Microemulsion System for Ocular Delivery of Sorafenib to Posterior Segment of the Eye

**DOI:** 10.3390/ijms22094404

**Published:** 2021-04-22

**Authors:** Manuela Santonocito, Cristina Zappulla, Santa Viola, Luca Rosario La Rosa, Elena Solfato, Ilenia Abbate, Valeria Tarallo, Ivana Apicella, Chiara Bianca Maria Platania, Grazia Maugeri, Velia D’Agata, Claudio Bucolo, Sandro De Falco, Maria Grazia Mazzone, Francesco Giuliano

**Affiliations:** 1 Research, Preclinical Development and Patents, SIFI S.p.A., Lavinaio-Aci S. Antonio, 95025 Catania, Italy; cristina.zappulla@sifigroup.com (C.Z.); santa.viola@sifigroup.com (S.V.); luca.larosa@sifigroup.com (L.R.L.R.); elena.solfato@sifigroup.com (E.S.); ilenia.abbate@sifigroup.com (I.A.); mariagrazia.mazzone@sifigroup.com (M.G.M.); francesco.giuliano@sifigroup.com (F.G.); 2 Institute of Genetics and Biophysics “Adriano Buzzati-Traverso”–CNR, 80131 Napoli, Italy; valeria.tarallo@igb.cnr.it (V.T.); apicella.ivana@gmail.com (I.A.); sandro.defalco@igb.cnr.it (S.D.F.); 3 Department of Biomedical and Biotechnological Sciences, School of Medicine, University of Catania, 95123 Catania, Italy; chiara.platania@unict.it (C.B.M.P.); grazia.maugeri@libero.it (G.M.); vdagata@unict.it (V.D.); claudio.bucolo@unict.it (C.B.)

**Keywords:** ocular drug delivery system, retina, angiogenesis, anti-VEGF, tyrosine kinase inhibitors, sorafenib, eye drops

## Abstract

Eye drop formulations allowing topical treatment of retinal pathologies have long been sought as alternatives to intravitreal administration. This study aimed to assess whether a novel nanostructured microemulsions system (NaMESys) could be usefully employed to deliver sorafenib to the retina following topical instillation. NaMESys carrying 0.3% sorafenib (NaMESys-SOR) proved to be cytocompatible in vitro on rabbit corneal cells, and well-tolerated following b.i.d. ocular administration to rabbits during a 3-month study. In rats subject to retinal ischemia-reperfusion, NaMESys-SOR significantly inhibited retinal expression of tumor necrosis factor-alpha (TNFα, 20.7%) and inducible nitric oxide synthase (iNos, 87.3%) mRNAs in comparison to controls. Similarly, in streptozotocin-induced diabetic rats, NaMESys-SOR inhibited retinal expression of nuclear factor kappa B (NFκB), TNFα, insulin like growth factor 1 (IGF1), IGF1 receptor (IGF1R), vascular endothelial growth factor receptor 1 (VEGFR1) and 2 (VEGFR2) mRNAs by three-fold on average compared to controls. Furthermore, a reduction in TNFα, VEGFR1 and VEGFR2 protein expression was observed by western blot. Moreover, in mice subject to laser-induced choroidal neovascularization, NaMESys-SOR significantly inhibited neovascular lesions by 54%. In conclusion, NaMESys-SOR was shown to be a well-tolerated ophthalmic formulation able to deliver effective amounts of sorafenib to the retina, reducing proinflammatory and pro-angiogenic mediators in reliable models of proliferative retinopathies. These findings warrant further investigations on the full therapeutic potential of NaMESys-SOR eye drops, aiming to address unmet needs in the pharmacotherapy of retinal neovascular diseases.

## 1. Introduction

Ocular neovascular diseases, including age-related macular degeneration (AMD), diabetic retinopathy (DR) and retinal vein occlusion (RVO), are the main causes of visual impairment worldwide leading to an irreversible degeneration of the macular region of the retina characterized by a progressive loss of photoreceptors and central vision [1]. In the foreseeable future, the incidence of these pathologies is expected to rise due to population aging and increasing life expectancy. Indeed, the number of total prevalent cases of AMD in nine of the major world economies (i.e., the US, China, Japan, Germany, the UK, France, Italy, Australia and Spain) is predicted to grow by about 18% by 2028 [2]. The estimated increase of diabetics by 2040 would lead to dramatic burden of care and socioeconomic impacts on the basis of current expensive treatment options and unavoidable indirect costs of DR-related blindness [3,4].

Pathological neoangiogenesis, that is the uncontrolled growth of new blood vessels from pre-existing vessels, and inflammation are common driving features of these vision-threatening diseases [5]. The aberrant angiogenesis is typically accompanied by fluid leakage and bleeding that lead to exudative or hemorrhagic retinal detachment [6,7]. The central role of vascular endothelial growth factor (VEGF) in the pathogenesis of a wide range of ocular neovascular diseases has been recognized for almost half a century [8,9,10,11].

Medical approaches to treatment of ocular neovascular diseases have greatly evolved in the past two decades, especially benefiting from the introduction of anti-VEGF agents. The availability of such therapeutic tools has redefined the standard of care for many retinal pathologies previously addressed mainly by means of ablative laser treatments [12].

Intravitreal treatment with anti-VEGF drugs has rapidly gained clinical relevance since the approval of pegaptanib, a 28-base ribonucleic acid aptamer, in 2004. Nowadays, drugs like ranibizumab, aflibercept and bevacizumab (the latter is off-label) are considered the first-line treatment in the fight against vision loss caused by retinal neovascular degeneration [13,14,15].

VEGF-based pharmacotherapy requires chronic treatment, potentially over many years, and is associated with many drawbacks including higher cost of newer agents, high frequency intravitreal injections, incomplete response and/or failure to maintain clinical response in some patients [16,17]. More importantly, administration of VEGF-blocking agents by repeated intravitreal injections may be associated with hypoxia, followed by tissue damage and other ocular complications and side-effects [18,19,20,21]. Indeed, along with therapies for dry AMD and geographic atrophy, and longer-acting anti-VEGF formulations/agents, less invasive routes of drug administration come as one of the most relevant unmet needs in the pharmacotherapy of degenerative retinal pathologies [2].

On this premise, we designed, developed and characterized a novel nanostructured microemulsions system (NaMESys) able to deliver pharmacologically levels of active ingredients to the retina following topical administration to the eye surface. Among several molecules shortlisted through bioinformatics-driven drug target prediction and drug repositioning, sorafenib tosylate was selected as a good candidate to be formulated and administered by means of NaMESys in the ocular setting. This choice was supported by the evidence that sorafenib is a well-known oral multi-kinase inhibitor currently approved for the anti-neovascular treatment of several cancer pathologies such as advanced renal cell carcinoma, hepatocellular carcinoma and differentiated thyroid cancer refractory to radioactive iodine [22,23]. Importantly, it is known that sorafenib inhibits VEGF and several tyrosine kinase receptors involved in tumor cell proliferation and tumor angiogenesis, including platelet-derived growth factor receptor-b (PDGF), which are factors known to be overexpressed in neovascular AMD [24,25]. Notably, the selection of sorafenib tosylate was also supported by a single-case study, reporting that sorafenib tosylate was able to exert remarkable therapeutic effects on the retina, following oral administration [26].

Here we sought to investigate the therapeutic potential of NaMESys eye drops loaded with 0.3% sorafenib tosylate (NaMESys-SOR). To this end, we carried out in vitro and in vivo biocompatibility tests as well as efficacy studies in widely used rodent models of retinal diseases, such as retinal ischemia-reperfusion (IR), diabetic retinopathy (DR) and laser-induced choroidal neovascularization (CNV) [27,28,29,30]. These studies provide insight on the safety and efficacy of NaMESys-SOR while contributing arguments in favor of the continued search for alternative paradigms to current pharmacotherapy of retinal diseases.

## 2. Results

### 2.1. Cytotoxicity of NaMESys-SOR on SIRC Cells

**Figure 1:**
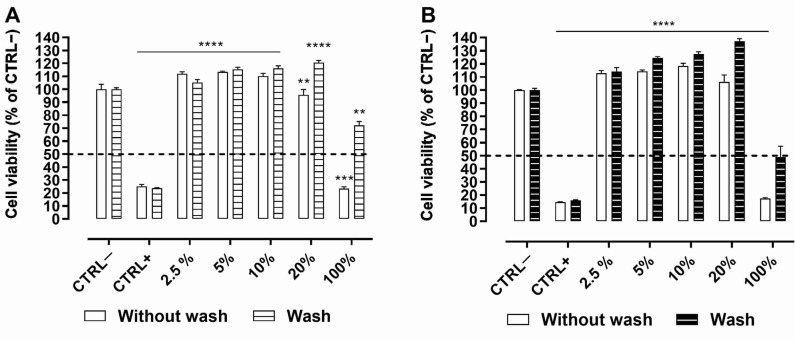
Cell viability of nanostructured microemulsions systems (NaMESys) in Statens Seruminstitut Rabbit Cornea (SIRC) cells by Short Time Repeated Exposure (S.T.R.E.) protocol. SIRC were repeatedly exposed 6 times at 1.5 h intervals between each repeated exposure for 5 min with or without wash to negative control (CTRL−), 0.01% BAK positive control (CTRL+), undiluted test items (100%) and to different dilutions of the same test items corresponding to 20%, 10%, 5% and 2.5% concentrations of (**A**) control formulation (NaMESys) or (**B**) microemulsion carrying sorafenib tosylate 0.3% (NaMESys-SOR). A dotted line placed at 50% represents the cut-off value to determine cytotoxicity potential according to ECVAM protocol DB-ALM n° 17. Data are presented as mean ± S.E.M. of two independent experiments performed in triplicate. ** *p* ≤ 0.01, *** *p* ≤ 0.001 and **** *p* ≤ 0.0001 vs. cut-off at 50%.

Statens Seruminstitut Rabbit Cornea (SIRC) cells were repeatedly exposed (6 times) to undiluted NaMESys or NaMESys-SOR (100%) or to different diluted concentrations of the same test items (20%; 10%; 5%; 2.5%) for 5 min with or without wash, with an interval of 1.5 h between the repeated exposures. Evaluation carried out by means of this Short Time Repeated Exposure (S.T.R.E.) protocol demonstrated that NaMESys (Figure 1A) and NaMESys-SOR (Figure 1B) were devoid of any significant cytotoxic effect at all concentrations tested both in “wash” and “no wash” conditions. Concerning the undiluted test items (100%), NaMESys and NaMESys-SOR produced cytotoxic effects exclusively in the very extreme “no wash” test condition, inducing cell mortality by 77% and 83%, respectively (Figure 1A,B). In contrast, the more realistic “wash” condition, which mimics lacrimal fluid turnover and drainage, was sufficient to restore cell viability of SIRC cells exposed to undiluted NaMESys and NaMESys-SOR (Figure 1A,B). Differently, benzalkonium chloride (BAK) 0.01% (CTRL+) induced significant cell mortality by 80–90% in all treatment conditions, causing the permanent loss of cell viability regardless of the “wash” or “no wash” condition (Figure 1A,B).

Overall, on the basis of in vitro evaluations, NaMESys and NaMESys-SOR were found to be cytocompatible and expected to be well tolerated by the eye surface in vivo.

### 2.2. Three-Month Ocular Tolerability Study in Rabbit

Ocular tolerability of NaMESys-SOR was evaluated versus NaMESys alone following monolateral instillation of 50 µL twice daily (b.i.d.) to the eyes of New Zealand White (NZW) rabbits for 91 days.

Ophthalmoscopic assessment and scoring according to Draize’s test demonstrated that treatments with NaMESys-SOR or NaMESys alone having only produced sporadic and sparse events of transient and low-score conjunctival redness, i.e., a maximum score of 1 in a scale from 0 to 3. In particular, on a total of 150 eye observations carried out on each treatment group during the whole study, only 3 and 11 instances of conjunctival redness were recorded for NaMESys-SOR and NaMESys, respectively. Consistently, in contralateral not treated eyes summing up to 300 observations, the same number of 11 instances of low-score redness were recorded. Differences among treatments and between treatments and respective controls were found to be statistically non-significant (*p* > 0.05, Chi-square test). Further observations carried out by slit-lamp weekly before administration of treatments, which strengthened these findings demonstrating that the redness described for a limited number of subjects was only transient. In particular, with the exception of one eye treated with NaMESys and two control eyes in both treatment groups, none of the study eyes was assigned a score for conjunctival redness in more than one occasion during the study. The slit-lamp examination also demonstrated that both treatments only produced a slight and transient fluorescein staining of the cornea confined to a small focus (score of 1 in a scale from 0 to 4 both for intensity and area), similarly to control not treated eyes. Specifically, out of total of 140 eye observations, only 6 and 8 instances of slight corneal staining were recorded for NaMESys-SOR and NaMESys, respectively. Control eyes only presented 5 instances of slight corneal staining out of 280 eyes examinations. Differences among treatments and between treatments and respective controls were found to be statistically non-significant (*p* > 0.05, Chi-square test). Similarly to evaluations according to the Draize scale, none of the study eyes was assigned a score for corneal staining in more than one occasion during the study.

Following histological analysis of ocular tissues and lids, no signs of damage related to the treatments were found in any of the eyes tested. All together, these data showed that both NaMESys-SOR and NaMESys were found to be macroscopically and microscopically well tolerated.

### 2.3. Efficacy of Topical NaMESys-SOR on Retinal IR in Rats

**Figure 2:**
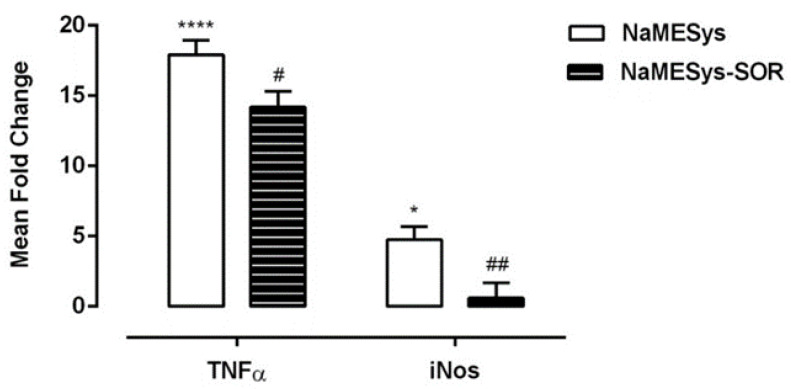
Quantitative real-time PCR gene expression profile of proinflammatory mediators tumor necrosis factor-alpha (TNFα) and inducible nitric oxide synthase (iNos) in ischemia-reperfusion (IR) rat retinas. Rats were pretreated topically b.i.d. 2 days and 1 h before inducing IR with microemulsions alone (NaMESys) or containing 0.3% sorafenib tosylate (NaMESys-SOR) and sacrificed 6 h after the ischemic episode. The data are presented as average ± SEM of the Fold Change in relation to the calibrator (NAÏVE CTRL). # *p* ≤ 0.05 and ## *p* ≤ 0.01 vs. NaMESys. * *p* ≤ 0.05 and **** *p* ≤ 0.0001 vs. NAÏVE CTRL. One-way ANOVA followed by Dunnett’s post-hoc test.

Quantitative real-time PCR analysis showed that 6 h after IR injury, the expression of both tumor necrosis factor-alpha (TNFα) and inducible nitric oxide synthase (iNos) mRNAs was strongly upregulated in the retina of rats topically treated b.i.d. for 2 days and 1 h before the ischemic insult with NaMESys with respect to naïve control used as the calibrator sample (Figure 2). Importantly, topical pretreatment with NaMESys-SOR significantly reduced the expression of TNFα (20.7%) and iNos (87.3%) (# *p* ≤ 0.05, ## *p* ≤ 0.01) compared to that observed in retinas of animals treated with vehicle alone (Figure 2).

Thus, sorafenib tosylate was effectively delivered to the back of the eye by NaMESys after topical instillation and was able to significantly downregulate the expression of inflammatory mediators that contribute to retinal damage in pathological conditions.

### 2.4. Efficacy Profile of NaMESys-SOR in the Streptozotocin (STZ)-Induced DR Model in Rats

Diabetes was induced in Sprague Dawley rats by i.p. injection of STZ (60 mg/kg); rats randomly assigned to non-diabetic control group were subjected to i.p. administration of citrate buffer. After induction of diabetes (24 h), verified by measurement of blood glucose levels to values above 250 mg/dL, diabetic rats were randomly assigned to the vehicle (NaMESys) or 0.3% sorafenib (NaMESys-SOR) treatment groups.

Specifically, animals were topically treated (12 µL) b.i.d. for 21 days and divided in three groups: non-diabetic control rats treated with vehicle (CTRL), diabetic rats treated with vehicle (NaMESys) and diabetic rats treated with microemulsion carrying 0.3% sorafenib tosylate (NaMESys-SOR).

**Figure 3:**
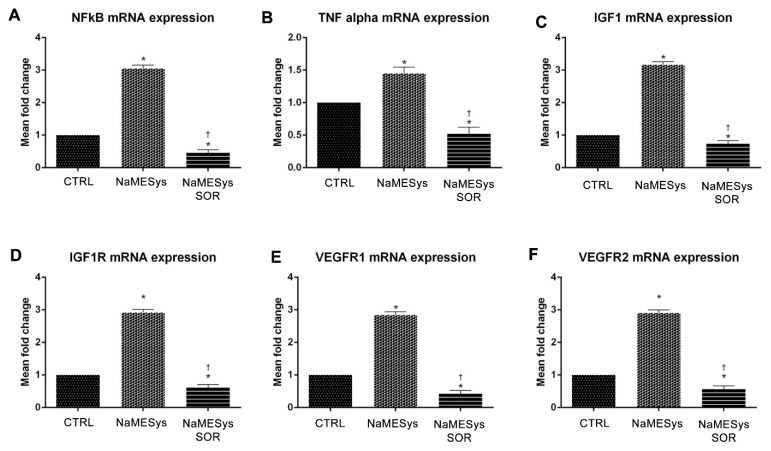
Expression of nuclear factor kappa B (NFκB), tumor necrosis factor-alpha (TNFα), insulin like growth factor 1 (IGF1), insulin like growth factor 1 receptor (IGFR1), vascular endothelial growth factor receptor 1 (VEGFR1) and vascular endothelial growth factor receptor 2 (VEGFR2) mRNA levels in retinas of diabetic rats treated with nanostructured microemulsions system carrying 0.3% sorafenib (NaMESys-SOR). Quantitative real-time PCR analysis of (**A**) NFκB, (**B**) TNFα, (**C**) IGF1, (**D**) IGFR1, (**E**) VEGFR1 and (**F**) VEGFR2 expression in retina of non-diabetic control rats treated with vehicle NaMESys (CTRL, *n* = 12), diabetic rats treated with vehicle (NaMESys, *n* = 12) and diabetic rats treated with microemulsion containing 0.3% sorafenib tosylate (NaMESys-SOR, *n* = 12). All treatments were administered b.i.d. topically (12 µL) for 21 days. mRNA expression of each gene (mean ± SD) was normalized to the endogenous ribosomal protein 18 S (housekeeping gene). Relative fold changes were calculated using the comparative ΔCt method. Expression levels were normalized to control. * *p* < 0.05 vs. CTRL or † *p* < 0.05 vs. NaMESys. One-way ANOVA followed by Tukey–Kramer’s post-hoc test.

Gene expression analysis by quantitative real-time PCR revealed that TNFα, nuclear factor kappa B (NFκB), vascular endothelial growth factor receptor 1 (VEGFR1) and 2 (VEGFR2), insulin like growth factor 1 (IGF1) and insulin like growth factor 1 receptor (IGF1R) mRNA levels increased significantly in the diabetic retina of NaMESys group compared to CTRL (Figure 3A–F). Interestingly, in NaMESys-SOR group the retinal expression of these proinflammatory and pro-angiogenic genes significantly decreased, compared to vehicle-treated diabetic rats (NaMESys), and even to vehicle-treated non-diabetic control rats (CTRL) (Figure 3A–F).

**Figure 4:**
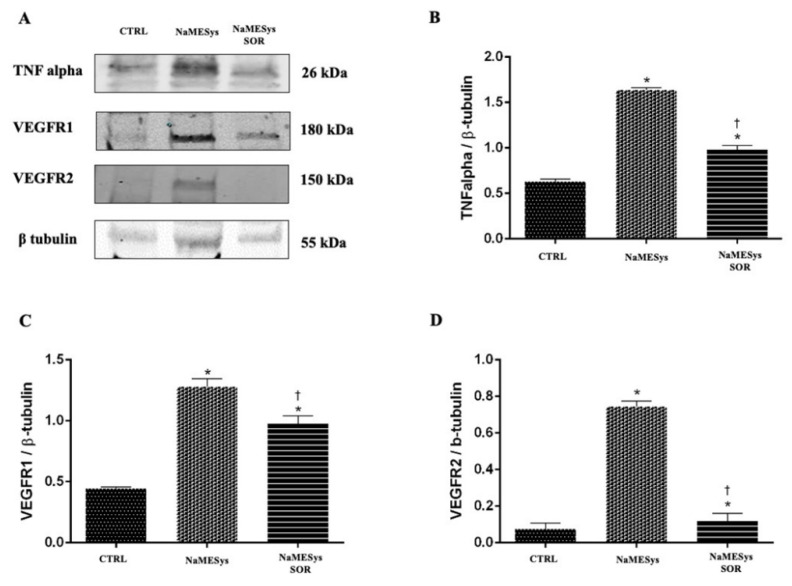
Tumor necrosis factor-alpha (TNFα), vascular endothelial growth factor receptor 1 (VEGFR1) and vascular endothelial growth factor receptor 2 (VEGFR2) protein expression in retinas of diabetic rats treated with nanostructured microemulsions system carrying 0.3% sorafenib (NaMESys-SOR). (**A**) Representative immunoblots of TNFα, VEGFR1 and VEGFR2 expression in retinas of control non-diabetic rats treated with vehicle NaMESys (CTRL, *n* = 12), diabetic rats treated with vehicle NaMESys (NaMESys, *n* = 12) and diabetic rats treated topically with NaMESys-SOR for 21 days (NaMESys-SOR, *n* = 12). Densitometric analyses of immunoblots for (**B**) TNF-α, (**C**) VEGFR1, (**D**) VEGFR2. Protein levels are expressed as arbitrary units obtained after normalization to β-tubulin which was used as loading control. Data are presented as mean ± SD. * *p* < 0.05 vs. CTRL or † *p* < 0.05 vs. NaMESys. One-way ANOVA followed by Tukey–Kramer’s post-hoc test.

Furthermore, we assessed retinal protein expression by means of Western Blot (WB) analyses. Specifically, the protein levels of inflammatory cytokine TNFα and of the two VEGF receptors, VEGFR1 and VEGFR2, were significantly expressed in the retinas of vehicle treated diabetic rats, compared to levels detected in retinas of non-diabetic control rats. Notably, topical ocular administration of NaMESys-SOR for 21 days significantly decreased the expression of these proteins, compared to the levels detected in the retinas of diabetic rats treated with the vehicle alone (Figure 4A–D).

These results confirmed the efficacy of NaMESys-SOR, which was able to deliver effective concentrations of sorafenib in the back of the eye, leading to significant anti-inflammatory and anti-angiogenic effects in diabetic retinas.

### 2.5. Efficacy of Topical NaMESys-SOR on Laser-Induced CNV in Mice

**Figure 5:**
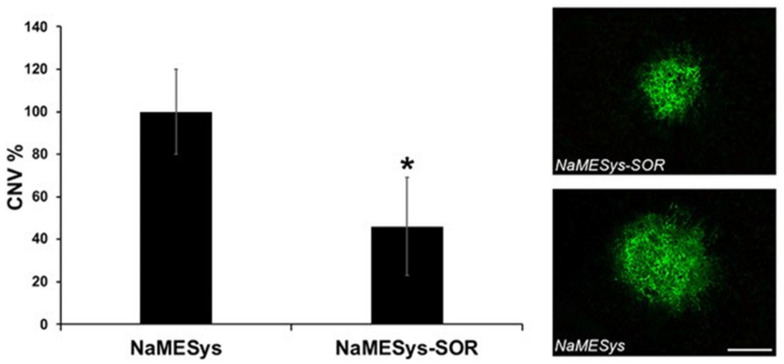
Nanostructured microemulsions system carrying 0.3% sorafenib (NaMESys-SOR) inhibited laser-induced choroidal neovascularization (CNV) when delivered by topical administration. CNV volumes were measured after 7 days from laser-induced damage by Isolectin B4 staining of retinal pigment epithelium (RPE)-choroid flat mounts. *n* = 5 mice per group. The following number of spots were analyzed: NaMESys = 14, NaMESys-SOR = 20. Data are presented as the mean ± SEM. * *p* = 0.0135 vs. vehicle (NaMESys). On the right, representative pictures of CNV. Scale bar: 100 µm.

Mice pre-treated b.i.d. for three days before laser induction and for the following 7 days with NaMESys-SOR showed a significant (*p* = 0.0135, 54%) reduction of CNV volume when compared to animals treated with NaMESys alone (Figure 5).

Overall, these results demonstrate that the microemulsion NaMESys is an effective drug delivery system, since it is able to carry sorafenib tosylate to the posterior segment of the eye inhibiting laser induced CNV.

## 3. Discussion

In the last decade, anti-VEGF agents have become the first-line therapy for ocular neovascular disorders. Although currently available treatments are effective at slowing vision loss, and improve vision for some patients, there are several practical limitations to consider. Indeed, anti-VEGF therapies require high cost for certain agents, careful follow up and frequent, chronic intraocular injections to achieve optimal efficacy, which is not reached in poor-responder subjects. In addition to the significant burden of care (direct and indirect costs), frequent intravitreal injections increase the risk of complications including endophthalmitis, temporary rise of intraocular pressure (IOP), cataract, retinal detachment and vitreous hemorrhage. Therefore, the necessity to overcome the burden of care associated with expensive treatments and indirect costs related to blindness has led to an increasing demand of new therapeutic strategies that fulfill the considerable unmet needs existing in the market [31]. Recent advancements in this field have focused on less invasive alternatives such as topical eye drops delivery of both small and large molecules to the back of the eye. Among these topical agents, promising drugs in early-stage development such as VEGF inhibitors (LHA510 Phase IIa, and PAN-90806 Phase I/II), integrin αvβ3 antagonist (SF0166, Phase I/II), and VEGF, PDGF and basic fibroblast growth factor inhibitors (squalamine lactate) have been investigated in clinical trials but none has been approved so far [32].

It is known that VEGF plays a pivotal role in both physiological and pathological angiogenesis and vascular permeability in retinal disorders by the activation of tyrosine-kinase receptors, and this makes VEGF the best target for therapeutic intervention [33,34,35].

In this study, we applied advances made in other therapeutic fields to ophthalmology by repurposing sorafenib tosylate, a well-known tyrosine-kinase inhibitor currently approved for the anti-neovascular treatment of several cancer pathologies (i.e., renal cell carcinoma, hepatocellular carcinoma) [22,23,36]. This choice was also supported by a single-case study reporting sorafenib tosylate as being able to exert measurable therapeutic effects on the retina following oral administration [26]. Moreover, scientific literature reports advances in the development of microemulsion formulations and nanoparticle systems designed to enhance the stability and residence time of the drugs, and to facilitate their deliver to the target site in several types of tumors [37]. Therefore, we designed, developed and characterized NaMESys, a novel nanostructured microemulsions system particularly suited for producing stable formulations of lipophilic molecules for ophthalmic delivery [38,39,40]. More importantly, NaMESys proved to be endowed with specific characteristics enabling the delivery of effective concentrations of small-molecule drugs to the retina following topical ocular administration to the eye surface. On this premise, we assessed ocular safety and efficacy of NaMESys-SOR by means of validated in vitro and in vivo biocompatibility test systems, as well as widely used retinal vascular pathology models in rodents.

Firstly, we demonstrated that NaMESys-SOR is well tolerated in vitro in SIRC cells using a predictive S.T.R.E. protocol. We devised it to account for the short contact times of active substances with the eye surface upon administration of eye drops. Notably, standard cytotoxicity protocols prescribe 24 h of cell exposure to test items [41], and this exposure time is rather extreme if compared to eye drops residence time on the human ocular surface of only a few minutes [42]. Certainly, this approach is most likely overestimating true cytotoxic effects. It is clear that the ocular surface is a very open system, the dynamics of which are difficult to reproduce in vitro. Takahashi et al. have devised a test strategy in order to overcome some of the limitations noted in existing cytotoxicity methods. In particular, they developed a protocol that requires a short time of exposure (i.e., 5 min) and demonstrated the need to dilute 1:20 the test items to be assessed in vitro to reach a 5% concentration, in order to produce reliable results consistent with known Draize data [43]. Therefore, we developed the S.T.R.E. test using SIRC cells as an alternative method for assessing eye irritation merging notion from both the DB-ALM Protocol n° 17 [41] and the short time exposure test by Takahashi et al. to better mimic the real situation after administration of drugs to the ocular surface. Thus, the protocol entails exposing the target cells to test items for 5 min for a total of six repetitions, summing up to 30 min of total exposure in a 12 h interval. Hence, we have simulated both realistic corneal residence times and repeated administration courses typically associated with ophthalmic eye drops treatments. NaMESys-SOR was found to be endowed with a particularly favorable profile when tested in the “wash” protocol. Importantly, this latter should be considered the most predictive condition of potential corneal cytotoxic effects arising from administration to the surface of the eye. The only cytotoxic effect on SIRC cells was shown exclusively for undiluted (100%) test items in the “no wash” condition and this gives rise to two main observations to take into account. First, this cytotoxicity is comparable to that of the positive control BAK 0.01%, which is the major preservative component currently used in eye drops at concentrations even higher than 0.01% (i.e., 0.02%) [44,45]. Second, unlike cytotoxicity results for BAK, the “wash” step was sufficient to restore cell viability of SIRC cells after treatment with either NaMESys or NaMESys-SOR. These findings further confirm that the “wash” condition is more predictive of what naturally occurs on the ocular surface after topical administration of eye drops [46]. Importantly, in our experiments, both formulations NaMESys and NaMESys-SOR at 5% concentration, the most reliable condition, were devoid of any cytotoxic effect either in a “wash” or “no wash” condition.

NaMESys-SOR also showed a good tolerability profile in NZW rabbits during a long-term ocular tolerance study. No relevant ocular pathological findings were recorded following a twice daily ocular topical administrations of NaMESys-SOR for up to 3 months. This study revealed that the delivery system under investigation was well tolerated by the ocular surface when given alone or loaded with 0.3% sorafenib tosylate. Indeed, the slight and transient corneal staining recorded in some study eyes was found not to be associated with treatments administered. Indeed, typically observed during long housing and treatment periods, the occurrence of whole or partial corneal staining may be related to physiological desquamation of the corneal epithelium naturally occurring in 10–20% of rabbits [47].

Thus, our study met its primary endpoint concerning its overall biocompatibility, with no cytotoxicity or ocular serious adverse events related to topical administration of NaMESys-SOR.

Because of several anatomical and physiological barriers in the anterior segment, a typical major challenge of ocular topical treatments is the delivery of effective concentrations of the active ingredient in the posterior segment of the eye. Preliminary bioavailability evaluations carried out in albino NZW rabbits [48] demonstrated that, after a single topical instillation of NaMESys-SOR, sorafenib can be detected in high amounts in retinal samples while being nearly undetectable in plasma. These data corroborate with the results obtained after the assessment of the efficacy profile of NaMESys-SOR (0.3% sorafenib tosylate). The topical ocular administration of this formulation led to effective delivery of sorafenib in the retina of rodents, leading to reduced retinal inflammation and angiogenesis in three validated experimental animal models of proliferative retinal diseases: IR, DR and AMD.

Inflammation is a key event involved in the pathogenesis and progression of ocular neovascular diseases [10]. In our experiments, we evaluated the effect of topical treatment with NaMESys-SOR eye drops on the expression of two key inflammatory mediators, TNFα and iNos, after retinal IR injury in rats, a validated animal model for studying retinal neuronal cell damage after ischemic insult [49,50,51]. Following IR, when compared to naïve controls, TNFα and iNos expression was found to be upregulated in the retina of rats treated with vehicle (NaMESys). Notably, ocular administration of NaMESys-SOR significantly reduced the expression of mRNA levels of these two inflammatory mediators, which contribute to retinal damage in pathological conditions [52,53].

NaMESys-SOR modulation of proinflammatory and pro-angiogenic factors was further investigated in a model of diabetic retinopathy induced by STZ. Topical ocular treatment with NaMESys-SOR significantly (*p* < 0.05) inhibited the expression, at transcriptional and post-transcriptional level, of upstream (NFκB) and downstream (TNFα) factors of inflammation, compared to levels detected in the retina of diabetic rats treated with NaMESys. Insulin signaling was found to be dysregulated in retina of diabetic rats, compared to non-diabetic control rats, and NaMESys-SOR eye drops decreased retinal mRNA levels of both IGF1 and its related receptor IGFR1. Specifically, according to the mechanism of action of sorafenib, NaMESys-SOR topical ocular treatment inhibited the expression of VEGFR1 and VEGFR2 both at transcriptional and post-transcriptional levels, compared to diabetic rats treated with using a vehicle. Inhibition of TNFα release, along with inhibition of VEGF signaling in diabetic retinopathy, has already been described in diabetic rats treated with intravitreal injection of aflibercept [54], likely involving the extracellular signal-regulated kinase (ERK) pathway. Indeed, NaMESys-SOR topical ocular administration was effectively exerted in the retina anti-angiogenic and anti-inflammatory activity. As regards as the modulatory effects of NaMESys-SOR on insulin signaling, our data confirm the tight link between the VEGF pathway and the IGF1-IGF1R pathway, which is involved in regulation of VEGF expression and angiogenesis both in experimental model of retinal neovascularization [55] and in proliferative diabetic retinopathy patients [56].

Consistent with its reported pharmacological profile in oncology, when delivered topically by means of NaMESys, sorafenib tosylate was also able to exert its well-described anti-angiogenic activity in a murine model of laser induced CNV, with a reduction of CNV volume of 50% with respect to vehicle controls. The effect observed in our study was found to be extremely promising from a therapeutical perspective.

## 4. Materials and Methods

### 4.1. Materials

SIRC cells (ATCC-CCL-60) were obtained from LGC Standards S.r.l. (Milan, Italy). Basal Medium Eagle (BME), gentamicin, penicillin-streptomycin, l-glutamine (l-glu), Trypsin–EDTA and Fetal Bovine Serum (FBS) were from Lonza (Basel, Switzerland). Reagent for 3-(4,5-dimethilthiazol-2-yl)-2,5-dipheniltetrazolium bromide (MTT) assay was purchased from Sigma-Aldrich S.r.l. (Milan, Italy). Dimethyl sulfoxide (DMSO) was from Supelco and was developed by Merk Life Science s.r.l. (Milan, Italy). BAK 50% was obtained from Novo Nordisk Pharmatech A/S (Køge, Denmark). NaMESys is a proprietary drug delivery system consisting of an oil-in-water microemulsion containing 24.2% (*w*/*v*) of lipids. Sorafenib tosylate was purchased from Hetero Corporate Industrial Estates (Telangana, India). Zoletil 50 + 50 mg/mL was from Virbac (Milan, Italy). Domitor 1 mg/mL was from Orion Pharma s.r.l. (Milan, Italy). Tropimil 0.5% was from Farmigea S.p.A. (Pisa, Italy). RNAlater storage stabilization solution was obtained from QIAGEN s.r.l. (Milan, Italy). TRIzol reagent, DNase I Amplification Grade, SuperScript II Reverse Transcriptase, Random Hexamer Primer, Oligo(dT)20 primers, dNTP Set 100 mM and SuperScript III Reverse Transcriptase were from Invitrogen by Thermo Fischer Scientific (Carlsbad, CA, USA). TaqMan gene expression assays for TNFα and iNos and real-time PCR master mix were from Applied Biosystems (Foster City, CA, USA). STZ was purchased from Sigma-Aldrich (St. Louis, MO, USA). Accu-Check Active was from Roche Diagnostic (Milan, Italy). Primers for the analysis of TNFα, NFκB, VEGFR1, VEGFR2, IGF1 and IGF1R genes by quantitative real-time PCR were from Life Technologies by Thermo Fisher Scientific (Monza, Italy). Protease inhibitor cocktail was from Roche Diagnostics (Monza, Italy). Quant-it Kit Protein Assay and Mini-PROTEAN TGX precast gels were purchased from BIO-RAD (Hercules, CA, USA). Anti-TNFα, anti-VEGFR1, anti-VEGFR2 antibodies and rabbit anti β-tubulin were from Santa Cruz Biotechnology (Santa Cruz, CA, USA). FITC–Griffonia simplicifolia Isolectin was from Vector Laboratories (CA, USA). Ketamine hydrochloride (Imalgene) and xylazine (Sedaxylan) were from Alcyon (Cherasco, Italy).

### 4.2. Cell Viability in SIRC Cells

SIRC cells were grown in a humidified 5% CO_2_ atmosphere at 37 °C in complete culture medium (CCM), made of BME containing 10% FBS, 100 U/mL of penicillin-streptomycin, 0.1 mg/mL gentamicin and 2 mM L-glu. Each well of a 96-well tissue culture plate was seeded with 40,000 cells in 100 µL of CCM. Cells were allowed to grow at 37 °C, 5% CO_2_ until subconfluent (70–90%) and then subjected to a new protocol based on S.T.R.E. of cells to the test items. We developed it by merging a notion from both DB-ALM Protocol n° 17 [41] and the short time exposure test developed by Takahashi et al. [43]. In detail, SIRC cells were repeatedly exposed 6 times for 5 min at 1.5 h intervals to negative control (sterile culture medium consisting of FBS-free BME, CTRL−), positive control (0.01% BAK, CTRL+), undiluted NaMESys or NaMESys-SOR (100%) and to different dilutions of the same test items, i.e., 1:5; 1:10; 1:20 and 1:40 corresponding to the following concentrations 20%, 10%, 5% and 2.5%, respectively. All dilutions were prepared using sterile FBS-free BME. Treatments were removed after 5 min of exposure and all cells were re-fed with CCM. Before re-feeding, only wells included in the “wash” protocol were washed once with BME (free of FBS, antibiotics and l-glu). At the end of repeated exposures, the medium was replaced with 100 µl of MTT solution (0.2 mg MTT/mL of CCM). Following a 30 min incubation, MTT formazan was extracted with 100 µL of 100% DMSO. The optical density of the samples obtained was read at 570 nm in a microplate spectrophotometer (SPECTRAFluor Plus, Tecan, Männedorf, Switzerland). Cell viability was calculated as a percentage of negative control. All samples were tested in two independent experiments performed in triplicate.

### 4.3. Ocular Long-Term Tolerability Study on NZW Rabbits

Twenty NZW rabbits, weighing approximately 2 kg, were used in this study. The study was conducted in accordance with Good Laboratory Practice (GLP) at the test facility IRIS PHARMA. All animals were treated according to the Directive 2010/63/UE European Convention for the Protection of Vertebrate Animals used for Experimental and Other Scientific Purposes and to the Association for Research in Vision and Ophthalmology (ARVO) Statement for the Use of Animals in Ophthalmic and Vision Research. All animals were housed individually in standard cages under identical temperature (18 °C  ±  3 °C), relative humidity (45–80%) and controlled enrichment conditions and exposed to a 12-h light and darkness cycle in continuously ventilated rooms (15–20 air volumes per h). They received a standard dry pellet diet and water ad libitum. The animals were randomly divided in two groups of 10 animals (5 males, 5 females) corresponding to two treatments: NaMESys and NaMESys-SOR. They were topically instilled twice daily (50 μL) in the right eye for 3 months while left eyes were left untreated and considered as controls. The general clinical signs of all animals such as body weight, general appearance and food consumption were observed and recorded following the schedule summarized in Table 1.

Two types of ocular examinations for clinical evaluations were conducted using an ophthalmoscope and a slit lamp, as scheduled in Table 1. Both eyes of each rabbit were examined under a light source for cornea, conjunctiva and iris adverse reaction scoring according to Draize’s scale [57]. Slit lamp examinations of both eyes were performed to assess cornea, conjunctiva, iris and the inner parts of the eye adverse reactions according to McDonald–Shadduck’s scale [58,59]. At the end of the experiment, animals were euthanized by an intravenous injection of overdosed pentobarbital following a sedation. At the end of the measurement period, blood (approximately 6 mL) was sampled from central artery of ears of all animals, collected in anticoagulant tubes (K3-EDTA) and centrifuged at 2000× *g* for 10 min at 4 °C. Two aliquots of 1 mL (at least) of plasma were sampled in appropriate tubes (1.5 mL Axygen tubes), snap-frozen and stored at −25 °C. Immediately after euthanasia, both eyeballs (including optic nerve, conjunctivae and extraocular muscles) and separately adnexa (upper and lower eyelids) were sampled, fixed in 0.8% Davidson solution for 24–28 h then processed appropriately for histopathological evaluation.

### 4.4. Rat Retinal IR Injury Model

Male Brown-Norway adult rats weighing approximately 200 g were obtained from Charles River (Calco, Italy). All the animals were treated according to the ARVO Statement for the Use of Animals in Ophthalmic and Vision Research and the Directive 2010/63/EU of the European Parliament and of the Council. Protocols were approved by the Italian Ministry of Health (authorization no. 390/2015-PR of 20 May 2015). The animals were fed on standard laboratory food and were allowed free access to water in an air-conditioned room with a 12 h light/12 h dark cycle, 50–70% humidity at 20–24 °C. The experimental procedure leading to IR injury was performed according to Andreeva et al. [60] with modifications as described here below. Before inducing the IR, rats were treated topically for 2 days (12 µL b.i.d., right eye) and 1 h before the insult (12 µL, right eye) with NaMESys (*n* = 7) or NaMESys-SOR (*n* = 8). Animals were anaesthetized by intraperitoneal injection of Zoletil (375 µL/kg) and Domitor (35 µL/kg) and, following mydriasis induced by topical administration of Tropimil 0.5%, the IR was induced by inserting a 30 gauge needle into the front chamber of the right eye, connected to a container of physiological saline solution. The saline solution reservoir was positioned at a height from the operating table that guaranteed hydrostatic pressure at the eye level of approximately 130 mmHg. Raising IOP to such levels to induce the ischemia was confirmed by the “whitening” of the back of the eye. After 45 min, the needle was removed to restore the retinal blood flow. 6 h after the ischemic episode, the animals were sacrificed by CO_2_ inhalation and retina explants stored in 50 µL RNAlater for further RNA extraction by TRIzol reagent. Total RNA (1 μg) was treated with DNase I Amplification Grade to eliminate possible genomic DNA contamination and reverse-transcribed into cDNA with SuperScript II Reverse Transcriptase and Random Hexamer Primer. Quantitative real-time PCR was performed in an AbiPrism 7000 thermal cycler (Life Technologies, Modena, Italy) using TaqMan gene expression assays for TNFα (Rn01525860_g1) and iNos (Rn00561646_m1) [60]. The fold change in gene expression was calculated according to the 2^−ΔΔCt^ method (Ct, cycle threshold) [61] using actin beta (Actb) (Rn00667869_m1) as the endogenous reference gene and the average ΔCt of naïve control samples (NAÏVE CTRL, not receiving IR) as a calibrator.

### 4.5. Rat STZ-Induced Diabetic Retinopathy Model

Male Sprague-Dawley rats (200–250 g) were obtained from Envigo (San Pietro a Nadisone, Udine, Italy). All the animals were treated according to the ARVO Statement for the Use of Animals in Ophthalmology and Vision Research and protocols were approved by the Italian Ministry of Health (authorization n. 1172/2016-PR). Animals were housed under standard conditions, with free access to water and standard chow, in a light-controlled (12 h light/dark cycle) room with standard temperature and humidity conditions. The induction of diabetes was performed by a single dose of STZ (60 mg/kg) that was intraperitoneally injected. After 24 h, the diabetic state of all animals was evaluated using a blood glucose meter and only animals with blood glucose levels greater than 250 mg/dL were considered diabetic. Diabetic rats body weight has been monitored during the study (no more than 10% body weight loss was observed in diabetic rats). Starting from the day when the glycemia was measured, 36 animals were randomly assigned to three experimental groups (*n* = 12 each group): (1) control group, treated with intraperitoneal injection of citrate buffer and topical administration of vehicle NaMESys (CTRL); (2) positive control group, injected with STZ (60 mg/kg, i.p.) and topically treated with vehicle (NaMESys); (3) treated group, injected with STZ (60 mg/kg, i.p.) and topically treated with microemulsion carrying 0.3% sorafenib tosylate (NaMESys-SOR). All animals received two daily administrations (12 µL/eye) NaMESys or NaMESys-SOR for 21 days, in both eyes. At the end of the treatment course, animals were sacrificed by CO_2_ inhalation, the eyes enucleated, and the retinas collected and stored at −80 °C until used for gene and protein analyses by quantitative real-time PCR and WB, respectively. In particular, 12 retinas per experimental group, from 36 animals, were processed for WB analyses, while the remaining 12 retinas per experimental group, from 36 animals, were processed for quantitative real-time PCR analyses. As regards quantitative real-time PCR analyses, retinal samples have been homogenized with TRIzol reagent in order to proceed with total RNA extraction. Purity and concentration of RNA extracts were assessed through photometric analysis with the BioPhotometer instrument (Eppendorf^®^). For each sample, 10 μg/μL mRNA wwas retrotranscribed using the following reagents: Oligo(dT)20 primers, dNTP Set 100 mM and the SuperScript^®^ III Reverse Transcriptase kit. Thereafter, 50 ng/μL cDNA for each sample were added of Applied Biosystems^®^ SYBR^®^ Green PCR Master Mix for the real-time PCR analysis by means of the Applied Biosystems^®^ 7500 Real-Time PCR Systems. The primers for target genes are hereby listed: TNFα forward: GAGCACGGAAAGCATGATCC and reverse: TAGACAGAAGAGCGTGGTGG; NFκB forward: CATCCACCTTCATGCTCAGC and reverse: TCCACCACATCTTCCTGCTT; VEGFR1 forward: AGGAAACAGAATCGAGGGCA and reverse: GCCTTGCAGCTGTAGATTCC; VEGFR2 forward: TTGGAAACTGAATGGCACCG and reverse: GCAGAGCAGACATAGTTGCC; IGF1 forward: TTCCGGAGCTGTGATCTGAG and reverse: TGAGTCTTGGGCATGTCAGT; IGF1R forward: TGTCCTCTCGGCATCAAACT and reverse: AGTAGTTGTGCCGGAACAGA. Gene expression was normalized using the S18 “housekeeping” gene. Results were analyzed with the 2^−ΔΔCt^ method. Quantitative real-time PCR adhered to the MiQE guidelines, and each sample has been run in triplicate. As regards the WB analysis, retinas were homogenized in a buffer containing 20 mM Tris (pH 7.4), 2 mM EDTA, 0.5 mM EGTA, 50 mM mercaptoethanol, 0.32 mM sucrose and a protease inhibitor cocktail, sonicated and centrifuged at 10,000 rpm for 10 min at 4 °C. Protein concentration was analyzed with the Quant-it Kit Protein Assay. For each sample, 40 µg of proteins was diluted with 2× Laemmli buffer and β-mercaptoethanol (20:1) and denaturated at 70 °C for 10 min. After that proteins were loaded in a mini-PROTEAN TGX precast gels and gel electrophoresis was carried out. Separated proteins have been transferred to a nitrocellulose membrane that was blocked with the Odissey blocking buffer (LI-COR) and incubated with specific primary antibody for anti-TNFα (sc-52746, 1:200), anti-VEGFR1 (Flt-1 (C-17) (sc-316, 1:500) and anti-VEGFR2 (Flk-1 (A-3) (sc-6251, 1:500). Immunoblots were normalized to β-tubulin expression (rabbit anti β-tubulin, sc-9104; 1:500). After overnight incubation with primary antibodies, membranes were washed with Tris-buffered saline-Tween20 (TBST) and then incubated with secondary antibodies anti-rabbit IRDye 800CW and anti-mouse IRDye 680 CW. Immunoblot detection was carried out with the Odyssey Infrared Imaging System (LI-COR; Lincoln, Nebraska, USA). Densitometric analysis was carried out with the ImageJ software (ImageJ software https://imagej.net/Welcome. Last accessed 1 April 2021).

### 4.6. Murine Model of CNV

C57Bl6/J 6–8 week-old male mice were purchased from Charles River (Calco, Italy). Animal experiments were in accordance with European directives no. 2010/63/UE and Italian directives D.lgs. 26/2014, and were approved by the Italian Ministry of Health (authorization n. 695/2015-PR of 17 July 2015).

Mice were pre-treated topically (5 μL b.i.d., both eyes) for three days preceding the induction of CNV and for the following 7 days with NaMESys (*n* = 5) or NaMESys-SOR (*n* = 5). Anesthesia was performed by intraperitoneal injection of 100 mg/kg ketamine hydrochloride and 10 mg/kg xylazine. Laser photocoagulation was performed using a 532-nm laser (Meridian) connected to the Micron IV apparatus (Phoenix Research Laboratories, Pleasanton, CA, USA).

Four radial spots were performed in both eyes at an equal distance from the optic nerve by laser impulse (532 nm; duration 100 ms; power 200 mW). The neovascularization area was determined by means of an immunofluorescence test. The animals were sacrificed by cervical dislocation and the enucleated eyes were put in 4% paraformaldehyde, then, the front segment of the eye was removed and the remaining part, called the ”eye-cup” (sclera, choroid, retinal pigment epithelium (RPE) and retina), was incubated in the presence of 0.7% FITC–Griffonia simplicifolia Isolectin for 16 h. The volume of each spot was obtained by fluorescence acquisition of a stack of images (20–25 frames, each with a thickness of 1 μm) along the z axis, from the upper surface to the deepest focal plane, in relation to the RPE cells. The fluorescent areas of each frame composing a single stack were measured with the program ImageJ (NIH, Bethesda, MD, USA) and added together, thus obtaining a neovascularization volume measurement. Results are expressed as mean ± standard error of the mean SEM.

### 4.7. Statistical Analyses

All statistical analyses were conducted using GraphPad Prism software version 6 (San Diego, CA, USA). In all statistical tests, the significance threshold was set at *p* < 0.05, and p-values were adjusted to correct for multiple comparisons when appropriate. Cell viability data were analyzed by one sample *t*-test (treatment vs cut-off) assuming a cut-off value for cytotoxicity of 50% as per ECVAM protocol DB-ALM n° 17. In the long-term tolerability study, differences among treatments and between treatments and respective controls were sought by Chi-square test. An ANOVA LSD test was done for body weight (absolute) from baseline to Day 92, between each group of the same sex for each time-point. Differences among treatments in the rat retinal IR injury model were sought by one-way ANOVA followed by Dunnett’s post-hoc test. Statistical analyses involving treatment groups, in the STZ-induced diabetic retinopathy paradigm, included one-way ANOVA followed by Tukey–Kramer post-hoc test for multiple comparison. Multiple comparisons were carried out only if F had a *p* < 0.05, and no significant variance inhomogeneity was found within analyzed groups. Regarding the CNV murine model, differences among groups were compared by Student’s *t* test (two-tailed).

## 5. Conclusions

Administration of drugs by means of alternative routes to intravitreal injection is one of the most relevant unmet need in the pharmacotherapy of degenerative retinal pathologies. The present study shows that NaMESys eye drops may represent an effective tool to deliver significant amounts of drugs to the retina. NaMESys loaded with sorafenib tosylate was proven to reduce inflammation and neo-angiogenesis elicited by cytokines and receptors that are key to the etiology and pathogenesis of neovascular retinal disorders. Thus, NaMESys-SOR may offer several advantages over available therapies for the treatment of proliferative retinal diseases such as AMD and DR. Lack of injection-related risks, simple self-administration and the therapeutic manageability typically offered by eye drops administration may well contribute to making NaMESys-SOR a fine addition to the medications arsenal available to ophthalmologists. Indeed, it is in view of an effective contribution in overcoming the burden associated to current therapies that future efforts aimed at evaluating safety and efficacy of NaMESys-SOR in a clinical setup are deemed worthwhile. More generally, by fostering the continued search for novel drug delivery technologies, we remain confident that topical delivery of drugs to the posterior segment of the eye may attract a renewed interest of the scientific and medical communities and provide the necessary momentum to effectively address a socially relevant unmet therapeutic need.

## 6. Patents

Patents resulting from the work reported in this manuscript: Solfato E. et al. Microemulsion compositions. Publication Number WO/2020/250252. Publication Date 17 December 2020. International Application No. PCT/IT2019/000048. International Filing Date 11 June 2019.

## Figures and Tables

**Table 1:** Schedule table of procedures, ocular examinations and sampling.

Study Date	Procedure	Ocular Examination	Sampling
Baseline	General clinical examination Food consumption BW ^1^	Slit-lamp Observation with an ophthalmoscope	-
Day 1	General clinical examination Food consumption Instillations	Observation with an ophthalmoscope after the first administration of the day (+1 h ± 6 min and just before the second administration)	-
Day 2 to Day 91	General clinical examination (daily) Food consumption (daily) BW (weekly) Instillations (daily)	Slit-lamp before the first administration of the day (weekly) Observation with an ophthalmoscope after the first administration of the day (+1 h ± 6 min and just before the second administration; weekly)	-
Day 92	General clinical examination BW Euthanasia	-	Blood sampling Both eyeballs and adnexa for histopathology evaluation

^1^ BW = Body Weight.

## Abbreviations

AMD	Age-related macular degeneration
ARVO	Association for Research in Vision and Ophthalmology
BAK	Benzalkonium chloride
BME	Basal medium eagle
BW	Body weight
CCM	Complete culture medium
CNV	Choroidal neovascularization
DMSO	Dimethyl sulfoxide
DR	Diabetic retinopathy
ERK	Extracellular signal-regulated kinase
FBS	Fetal bovine serum
GLP	Good laboratory practice
IGF1	Insulin like growth factor 1
IGF1R	Insulin like growth factor 1 receptor
iNos	Inducible nitric oxide synthase
IOP	Intraocular pressure
IR	Ischemia-reperfusion
l-glu	l-glutamine
MTT	3-(4,5-dimethilthiazol-2-yl)-2,5-dipheniltetrazolium bromide
NaMESys	Nanostructured microemulsions system
NaMESys-SOR	Nanostructured microemulsions system carrying 0.3% sorafenib tosylate
NFκB	Nuclear factor kappa B
NZW	New zealand white
PDGF	Platelet-derived growth factor receptor-b
RVO	Retinal vein occlusion
S.T.R.E.	Short time repeated exposure
SIRC	Statens seruminstitut rabbit cornea
STZ	Streptozotocin
TNFα	Tumor necrosis factor-alpha
VEGF	Vascular endothelial growth factor
VEGFR1	Vascular endothelial growth factor receptor 1
VEGFR2	Vascular endothelial growth factor receptor 2
WB	Western blot
